# The effects of kisspeptin on β‐cell function, serum metabolites and appetite in humans

**DOI:** 10.1111/dom.13460

**Published:** 2018-08-16

**Authors:** Chioma Izzi‐Engbeaya, Alexander N. Comninos, Sophie A. Clarke, Anne Jomard, Lisa Yang, Sophie Jones, Ali Abbara, Shakunthala Narayanaswamy, Pei Chia Eng, Deborah Papadopoulou, Julia K. Prague, Paul Bech, Ian F. Godsland, Paul Bassett, Caroline Sands, Stephane Camuzeaux, Maria Gomez‐Romero, Jake T. M. Pearce, Matthew R. Lewis, Elaine Holmes, Jeremy K. Nicholson, Tricia Tan, Risheka Ratnasabapathy, Ming Hu, Gaelle Carrat, Lorenzo Piemonti, Marco Bugliani, Piero Marchetti, Paul R. Johnson, Stephen J. Hughes, A. M. James Shapiro, Guy A. Rutter, Waljit S. Dhillo

**Affiliations:** ^1^ Section of Endocrinology and Investigative Medicine, Division of Diabetes, Endocrinology and Metabolism, Department of Medicine Imperial College London London UK; ^2^ Department of Endocrinology Imperial College Healthcare NHS Trust London UK; ^3^ Section of Metabolic Medicine, Department of Medicine, Imperial College London St Mary's Hospital London UK; ^4^ Statsconsultancy Ltd Amersham UK; ^5^ The MRC‐NIHR National Phenome Centre and Imperial BRC Clinical Phenotyping Centre, Division of Computational, Systems and Digestive Medicine, Department of Surgery and Cancer London UK; ^6^ Section of Cell Biology and Functional Genomics, Division of Diabetes, Endocrinology and Metabolism, Department of Medicine Imperial College London London UK; ^7^ Imperial Pancreatic Islet Biology and Diabetes Consortium Hammersmith Hospital, Imperial College London London UK; ^8^ Diabetes Research Institute (SR‐DRI), IRCCS San Raffaele Scientific Institute Milan Italy; ^9^ Faculty of Medicine Vita‐Salute San Raffaele University Milan Italy; ^10^ Department of Clinical and Experimental Medicine, Islet Cell Laboratory University of Pisa Pisa Italy; ^11^ Nuffield Department of Surgical Sciences University of Oxford Oxford UK; ^12^ Oxford Centre for Diabetes, Endocrinology, and Metabolism University of Oxford Oxford UK; ^13^ National Institute of Health Research Oxford Biomedical Research Centre, Churchill Hospital Oxford UK; ^14^ Clinical Islet Laboratory and Clinical Islet Transplant Program University of Alberta Edmonton Canada

**Keywords:** appetite control, beta cell function, glucose metabolism, incretins, insulin secretion, islets

## Abstract

**Aims:**

To investigate the effect of kisspeptin on glucose‐stimulated insulin secretion and appetite in humans.

**Materials and methods:**

In 15 healthy men (age: 25.2 ± 1.1 years; BMI: 22.3 ± 0.5 kg m^−2^), we compared the effects of 1 nmol kg^−1^ h^−1^ kisspeptin versus vehicle administration on glucose‐stimulated insulin secretion, metabolites, gut hormones, appetite and food intake. In addition, we assessed the effect of kisspeptin on glucose‐stimulated insulin secretion in vitro in human pancreatic islets and a human β‐cell line (EndoC‐βH1 cells).

**Results:**

Kisspeptin administration to healthy men enhanced insulin secretion following an intravenous glucose load, and modulated serum metabolites. In keeping with this, kisspeptin increased glucose‐stimulated insulin secretion from human islets and a human pancreatic cell line in vitro. In addition, kisspeptin administration did not alter gut hormones, appetite or food intake in healthy men.

**Conclusions:**

Collectively, these data demonstrate for the first time a beneficial role for kisspeptin in insulin secretion in humans in vivo. This has important implications for our understanding of the links between reproduction and metabolism in humans, as well as for the ongoing translational development of kisspeptin‐based therapies for reproductive and potentially metabolic conditions.

## 1 INTRODUCTION

Metabolism and reproduction are fundamental aspects of mammalian physiology which are intricately linked. However, our understanding of the hormonal links between these two biological systems is limited. Recent evidence in animals suggests that the recently discovered hormone kisspeptin may link reproduction and metabolism; however, until now human in vivo studies have not been performed.

Kisspeptin sits at the apex of the hypothalamo‐pituitary‐gonadal axis, controlling downstream reproductive hormone secretion. Kisspeptin has pivotal roles in fertility,^1^ which are being utilised by kisspeptin‐based therapies now in development for common reproductive disorders.^2^, ^3^, ^4^, ^5^ Kisspeptin (*kiss1*) and its receptor (*kiss1r*) are expressed in the hypothalamus, pancreatic β‐cells, liver and adipose tissue,^6^, ^7^, ^8^, ^9^ suggesting roles in key autocrine and paracrine metabolic processes. Kisspeptin has recently been reported to increase glucose‐stimulated insulin secretion (GSIS) in rats^10^ and monkeys,^11^ and this is supported by in vitro islet studies which demonstrate enhancement of GSIS by kisspeptin.^8^, ^10^, ^12^, ^13^ Interestingly, it has also been reported that kisspeptin can inhibit GSIS at lower kisspeptin concentrations^13^ and low glucose concentrations,^14^ but stimulate GSIS at high kisspeptin concentrations.^13^

There are neuroanatomical and functional connections between kisspeptin and important hypothalamic appetite‐regulating neuropeptides.^15^, ^16^, ^17^ Intracerebroventricular administration of kisspeptin has been reported to alter food intake in mice^18^ and chicks,^19^ while other studies report no effect of kisspeptin administration on appetite in rats.^20^ In addition, impaired kisspeptin signalling disrupts metabolism and promotes glucose intolerance and obesity in mice.^21^

Thus, data in animals strongly suggests that kisspeptin plays a role in metabolism, but the effects of kisspeptin on metabolic parameters in vivo in humans are currently unknown. There is therefore an important clinical need to elucidate the metabolic effects of kisspeptin in humans, to further our understanding of the physiology of human metabolism, as well as to inform the ongoing development of kisspeptin‐based therapies.

In this study, we investigate the effects of kisspeptin on β‐cell function, metabolites and appetite in humans. We demonstrate that in humans, administration of kisspeptin stimulates GSIS in vivo and in vitro, and significantly modulates the concentrations of metabolites, some of which are known to be associated with insulin secretion. Furthermore, kisspeptin administration did not affect appetite or food intake. These data provide the first human in vivo insights into the metabolic actions of kisspeptin and have significant translational implications relating to glucose homeostasis.

## 2 MATERIALS AND METHODS

### 2.1 Human studies

#### 2.1.1 Study participants

This study was reviewed and approved by the West London Research Ethics Committee (16/LO/0391), and was performed in accordance with the Declaration of Helsinki. Healthy men (aged 18‐40 years) were recruited by online and print advertisements. Written informed consent was obtained from each participant prior to study enrolment. Exclusion criteria included body mass index (BMI) < 18.5 or > 25 kg m^−2^, a history of medical and psychological conditions, use of prescription, recreational or investigational drugs within the preceding 2 months, blood donation within 3 months of study participation, ingestion or inhalation of nicotine‐containing substances, alcoholism, and history of cancer.

Participants were instructed to abstain from strenuous exercise, alcohol and caffeine for 24 hours preceding each study visit. Each participant was instructed to choose a meal and eat that same meal at 8 pm on the night preceding each study visit, fast overnight, and attend the study visit fasted. Each participant underwent two intravenous glucose tolerance tests (IVGTTs, one with kisspeptin and one with vehicle administration) and/or two mixed meal tolerance tests (MMTTs, one with kisspeptin and one with vehicle administration).

#### 2.1.2 Infusions

Infusion order was randomized using a random number generator, and participants were blinded as to the identity of the infusion. Kisspeptin infusions were prepared by dissolving kisspeptin‐54 (Bachem, St Helens, uk) in 1 mL of 0.9% NaCl (Braun, Sheffield, UK) then adding the kisspeptin solution to 49 mL Gelofusine (Braun). Kisspeptin was infused at a rate of 1 nmol kg^−1^ h^−1^, a dose previously established to be bioactive in humans.^5^ Vehicle infusions consisted of Gelofusine administered at the equivalent rate to the kisspeptin infusion for each participant.

#### 2.1.3 IVGTT

**Figure 1:**
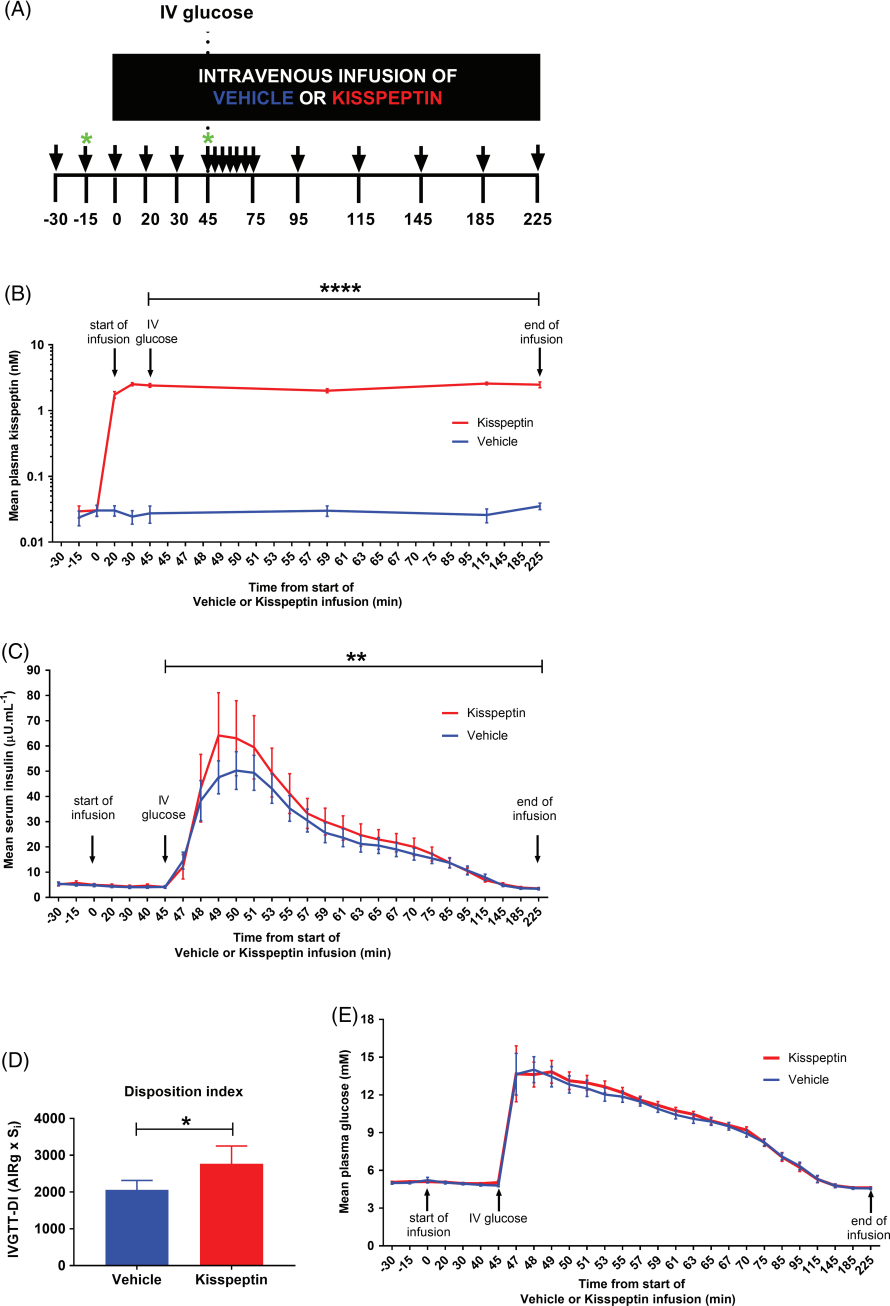
Kisspeptin administration enhances β‐cell function during IVGTT in humans. A, Following an overnight fast, 15 healthy men (age: 25.2 ± 1.1 years; BMI: 22.3 ± 0.5 kg M^−2^) were administered 1 nmol kg^−1^ hr^−1^ kisspeptin or rate‐matched vehicle for 225 minutes, in random order. At T = 45 minutes, 0.3 g kg^−1^ of 20% dextrose was infused intravenously over 2 minutes. Blood sampling was performed regularly (black arrows), with samples for metabolic profiling collected at T = −15 minutes and T = 45 minutes (green stars). B, Mean plasma kisspeptin levels during IVGTT were higher during kisspeptin infusion compared with vehicle administration as expected; **** *P* < 0.0001 kisspeptin vs. vehicle using GEE. C, Mean serum insulin levels during IVGTT were higher during kisspeptin infusion compared with vehicle administration; ** *P* = 0.01 kisspeptin vs. vehicle (multi‐level linear regression). D, Mean IVGTT‐DI was higher during kisspeptin administration (red bar) compared with vehicle administration (blue bar); * *P* < 0.05 kisspeptin vs. vehicle (paired t‐test). E, Mean plasma glucose levels during IVGTT were similar during kisspeptin and vehicle administration; *P* = 0.64 kisspeptin vs. vehicle (multi‐level linear regression). Data are presented as mean ± SEM; n = 15 per group

On arrival at the Clinical Research Facility (CRF), and after a period of acclimatisation, two intravenous cannulae (one in each antecubital fossa) were inserted (one for blood sampling and the other for intravenous infusion administration). Following baseline sampling, kisspeptin or vehicle infusion was started at T = 0 minutes and infused until T = 225 minutes. Next, 0.3 g kg^−1^ of 20% dextrose (Hameln, Gloucester, UK) was administered intravenously over 120 seconds starting from T = 45 minutes (i.e. when kisspeptin levels had reached a steady state, Figure 1A,B). To obtain the glucose and insulin values required for calculation of the acute insulin response to glucose (AIRg) and minimal model insulin sensitivity index (S_i_), an established frequent sampling protocol^22^ was used (Figure 1A). AIRg was calculated as the incremental AUC (using the trapezoid rule^23^) of insulin from T = 45 minutes to T = 55 minutes (i.e. 0‐10 minutes post‐glucose load). S_i_ was determined using the minimal model (MLAB software)^24^ and the disposition index (IVGTT‐DI) was calculated as the product of AIRg and S_i._ 22 Blood was taken for measurement of glucose, insulin, kisspeptin, luteinising hormone (LH), testosterone, glucagon‐like peptide‐1 (GLP‐1), peptide‐YY (PYY), glucagon and cortisol.

#### 2.1.4 MMTT

A taste test was performed prior to the MMTT study visits to determine the study meal (spaghetti bolognese [125 kcal 100 g^−1^] or mushroom risotto [124 kcal 100 g^−1^]; both from Waitrose, Bracknell, UK) for each participant. These meal options were selected as they provided similar kcal weight^−1^. Following acclimatisation and baseline sampling, kisspeptin or vehicle infusion was started at T = 0 minutes and continued until T = 120 minutes.

Visual analogue scales, to measure participants' self‐reported hunger, were performed at T = −30 minutes, T = 30 minutes and T = 75 minutes. Next, 45 minutes into the infusion (i.e. when kisspeptin levels had reached a steady state), an ad libitum meal was given to participants. They were instructed to eat until comfortably full; all clocks, electronic devices and reading material were taken away while the participants were eating, and researchers left the room (to minimize distractions which are known to influence meal size and duration). Blood was taken for the measurement of glucose, insulin, kisspeptin, LH and testosterone.

Calculations were made using established methods as follows: MMTT‐ISI (MMTT insulin secretion index) as the ratio of AUC insulin to AUC glucose, MMTT‐IS (MMTT insulin sensitivity index) using the Matsuda index (with T = 45 minutes values as baseline, and mean insulin and glucose values from T = 65‐120 minutes used in the equation), and MMTT‐DI (MMTT disposition index) as the product of MMTT‐ISI and MMTT‐IS.^25^

#### 2.1.5 Serum metabolite identification and analyses

Blood samples for metabolic profiling were taken pre‐infusion (T = −15 minutes) and at steady state prior to intravenous glucose or meal ingestion (T = 45 minutes). Serum sample handling (sorting and formatting) was performed as previously described,^26^ and 50 μL aliquots were taken from each sample for metabolite analyses. Protein was removed from the samples prior to analysis by addition of organic solvent, mixing and centrifugation, yielding a homogenous supernatant (method‐specific details are included in the supporting information for this article). The prepared samples were subjected to small molecule and lipid analyses by ultra‐performance liquid chromatography mass spectrometry (UPLC‐MS). Reversed‐phase chromatography tailored for complex lipid retention and separation was used to profile the lipid species of each sample, while hydrophilic interaction liquid chromatography (HILIC) was used to retain and separate polar metabolites.^26^ High resolution orthogonal acceleration time‐of‐flight mass spectrometry (oaTOF‐MS) operating in the positive ion mode was used for both assays.

All UPLC‐MS analyses were performed on Acquity UPLC instruments, coupled to Xevo G2‐S TOF mass spectrometers (Waters Corp., Manchester, UK) via a Z‐spray electrospray ionisation (ESI) source. Further details of the analytical methods used can be found in the supporting information for this article. Feature extraction and retention time alignment were performed in Progenesis QI (Waters Corp., Milford, MA). In‐house scripts (Python software) were developed for the elimination of potential run‐order effects and noise filtering. Linear mixed effect models were generated using the lmer4 R software package^27^ according to the formula: model <− Feature ~ Time*Class + (1|SubjectID) + (1|Challenge). A model was generated for each feature including fixed effects for interaction between class (kisspeptin or vehicle alone) and time (T = −15 minute and T = 45 minute), allowing for both participant and challenge‐specific random effects (owing to the presence of multiple challenges per participant). Features showing significant differences between the two timepoints for vehicle and/or kisspeptin classes were identified by false discovery rate correction of the appropriate model estimates using the locfdr package.^28^ Subsequently, assignment of significant features in the kisspeptin class alone (FDR α = 0.05) was performed. Where significant features were found to be adduct or fragment ions, the full spectrum (including the protonated molecule) was deduced and used in molecular assignment. Chemical identity was assigned by matching accurate mass and tandem mass spectrometry (MS/MS) fragmentation (of the protonated molecule) measurements to reference spectra using LIPID MAPS online tools (for lipid species)^29^ or an in‐house database constructed from analysis of authentic reference materials. Where authentic reference materials were commercially available, they were used to generate definitive molecular identification by direct matching of chromatographic and spectral qualities (including accurate mass, MS/MS spectra, and isotopic distribution) to those observed in the profiling data and subsequent targeted MS/MS experiments.

#### 2.1.6 Biochemical analyses

Plasma kisspeptin and gut hormone levels were measured using established in‐house radioimmunoassays (kisspeptin intra‐assay CV 8.3% and inter‐assay CV 10.2%, GLP‐1 intra‐assay and inter‐assay CV: ≤10%, PYY intra‐assay and inter‐assay CV: ≤10%, glucagon intra‐assay and inter‐assay CV: ≤10%).^5^, ^22^, ^30^ Serum insulin, plasma glucose, serum LH, serum testosterone and serum cortisol were measured in the Clinical Chemistry Laboratory of Imperial College Healthcare NHS Trust on the automated Abbott Architect® platform. Chemiluminescent immunoassays were used to measure serum insulin (intra‐assay and inter‐assay CV: ≤7%), serum LH (intra‐assay and inter‐assay CV: ≤5%), serum testosterone (intra‐assay and inter‐assay CV: ≤8%) and serum cortisol (intra‐assay and inter‐assay CV: ≤10%). Plasma glucose was measured using a colorimetric hexokinase assay (intra‐assay and inter‐assay CV: ≤2%).

### 2.2 In vitro studies

#### 2.2.1 Human islet cell culture

Preparations from six different donors (Table S1 in File S1) were used to perform insulin secretion experiments. Islets (10/well) were incubated in triplicate for each condition in a 12‐well non‐treated cell culture plate. Insulin secretion assays were performed in Krebs‐Ringer‐Hepes‐Bicarbonate (KRHB) buffer (10 mM Hepes, 2 mM NaHCO_3_, 140 mM NaCl, 3.6 mM KCl, 0.5 mM MgSO_4_, 0.5 mM NaH_2_PO_4_, 1.5 mM CaCl_2_ supplemented with 0.1% BSA) saturated with 95% O_2_/5% CO_2_ and adjusted to pH 7.4.^31^ Islets were pre‐incubated in a 37 °C water bath under agitation for 1 hour in 3 mM glucose KRHB prior to the secretion assay (for 30 minutes) in KRHB, 3 mM or 17 mM glucose, in the presence of different concentrations of kisspeptin‐54 (0, 2.7 or 1000 nM). The 2.7 nM dose was selected as this is similar to the plasma kisspeptin levels produced by 1 nmol.kg^−1^ h^−1^ kisspeptin administration in our in vivo study (i.e. 2‐3 nM during kisspeptin steady state – see Figure 1B). The 1000 nM dose was selected as this was similar to the dose of kisspeptin used in other in vitro studies.^8^, ^10^, ^13^ The supernatant was collected and the islets were lysed in 1 mL of acidified ethanol (75% ethanol, 23.5% H_2_O, 1.5% 1 M HCl, 0.1% Triton) and sonicated two times for 10 seconds, to extract total islet insulin content. Insulin concentration was measured using an ultrasensitive HTRF kit (Cisbio Bioassays, Codolet, France), and secreted insulin was normalized as percentage of total insulin content.

#### 2.2.2 Human β‐cell line (EndoC‐βH1 cells)

EndoC‐βH1 cells were seeded onto ECM‐coated 48‐well plates at 250 × 10^3^ cells per well. Four days after seeding, cells were incubated overnight in a glucose starving medium (glucose‐free DMEM‐2% albumin from bovine serum fraction V, 50 μL 2‐mercaptoethanol, 10 mM nicotinamide, 5.5 μg mL^−1^ transferrin, 6.7 ng mL^−1^ sodium selenite and penicillin/streptomycin) with glucose added to give a final concentration of 2.8 mM glucose. The next morning cells were incubated for 1 hour with Krebs Ringer solution (0.2% BSA, 25% solution 1, 25% solution II, 25% solution III, 10 mM Hepes) supplemented with 15 mM glucose. EndoC‐βH1 cells were then incubated in the presence of different concentrations of kisspeptin‐54 (0, 100 or 1000 nM). These doses are similar to those used in published in vitro studies.^8^, ^10^, ^13^ The cells were incubated in triplicate for each condition. After incubation for 60 minutes, the supernatant was collected, placed onto ice and centrifuged for 5 minutes at 3000 rpm at 4 °C. The supernatant was then transferred into a fresh tube. Cells were lysed in 50 μL of cell lysis solution (TETG: 20 mM Tris PH 8.0, 1% Triton X‐100, 10% glycerol, 137 mM NaCl, 2 mM EGTA). The lysate was then removed and placed onto ice, and centrifuged at 3000 rpm for 5 minutes at 4 °C. Insulin concentration was measured using an ultrasensitive HTRF kit (Cisbio Bioassays), and secreted insulin was normalized as percentage of total insulin content.

### 2.3 Statistical methods

Using STATA, an a priori power calculation was performed using a dataset of IVGTTs performed in 99 healthy men aged 18‐40 years, provided by I.F.G. Using this dataset, a study consisting of IVGTTs performed in 15 healthy men would have 80% power to detect a 25% difference in insulin secretion.

Statistical analyses were performed with the assistance of a statistician (PBa). Unless otherwise stated, statistical analysis was performed using Prism (GraphPad, CA) and data are presented as mean ± SEM. Paired t‐tests were performed on parametric data and Wilcoxon matched‐pairs signed rank tests were performed on non‐parametric data. Two‐way ANOVA with Dunnett's multiple comparison tests was performed for comparison of >2 groups of parametric data, while a Friedman test with Dunn's multiple comparison tests was performed for comparison of >2 groups of non‐parametric data. Multi‐level linear regression modelling was performed on insulin and glucose curves. The generalized estimating equation (GEE) was performed on other non‐independent longitudinal data using STATA (Statacorp, TX). Statistical significance was set at *P* < 0.05.

## 3 RESULTS

### 3.1 Elevated circulating kisspeptin enhances insulin secretion during intravenous glucose challenges in healthy men

To establish the effect of kisspeptin administration on GSIS in vivo in humans, 1 nmol kg^−1^ h^−1^ kisspeptin infusion (a dose of kisspeptin that has previously been shown to be bioactive in humans^5^), or rate‐matched vehicle infusion, was administered to 15 healthy young men in a randomized blinded two‐way crossover study. Using a well‐established frequently sampled IVGTT protocol^22^ (Figure 1A), a 0.3 g kg^−1^ intravenous glucose load was administered at T = 45 minutes, when circulating kisspeptin levels had reached steady state (Figure 1B). In response to the intravenous glucose load, kisspeptin administration significantly enhanced GSIS compared with vehicle (Figure 1C). Two‐level linear regression modelling confirmed that mean post‐glucose load insulin levels were 4.1 μU mL^−1^ higher (95% CI: 0.9‐7.3; p = 0.01) during kisspeptin compared with vehicle administration. The insulin sensitivity index (S_i_) (S_i_: kisspeptin 8.11 ± 0.98 min^−1^ mU^−1^ L.10^4^ vs. vehicle 6.85 ± 0.89 min^−1^ mU^−1^ L 10,^4^ p = 0.1228) was similar between groups.

Furthermore, kisspeptin elicited a significantly higher disposition index (IVGTT‐DI) compared with vehicle (IVGTT‐DI: kisspeptin 2768 ± 484 units vs. vehicle 2061 ± 255 units, p < 0.05, Figure 1D). The disposition index is a well‐validated method for assessing β‐cell function^32^ as it is comprised of measures of insulin secretion, insulin sensitivity and prevailing glucose concentrations (which were similar between groups; Figure 1E).

Kisspeptin administration resulted in elevated circulating kisspeptin levels (Figure 1B), which, as expected, resulted in elevated LH levels, confirming peptide bioactivity (Figure S1A). Consistent with previous studies,^5^, ^33^ testosterone levels did not rise during the time‐period of kisspeptin administration (Figure S1B).

Kisspeptin administration did not alter the circulating levels of endogenous insulin secretagogues GLP‐1 (Figure S1C), PYY (Figure S1D) and glucagon during IVGTT (Figure S1E). Additionally, kisspeptin administration did not affect circulating levels of cortisol, a counter‐regulatory hormone (Figure S1F).

### 3.2 Kisspeptin dose‐dependently stimulates GSIS in vitro

**Figure 2:**
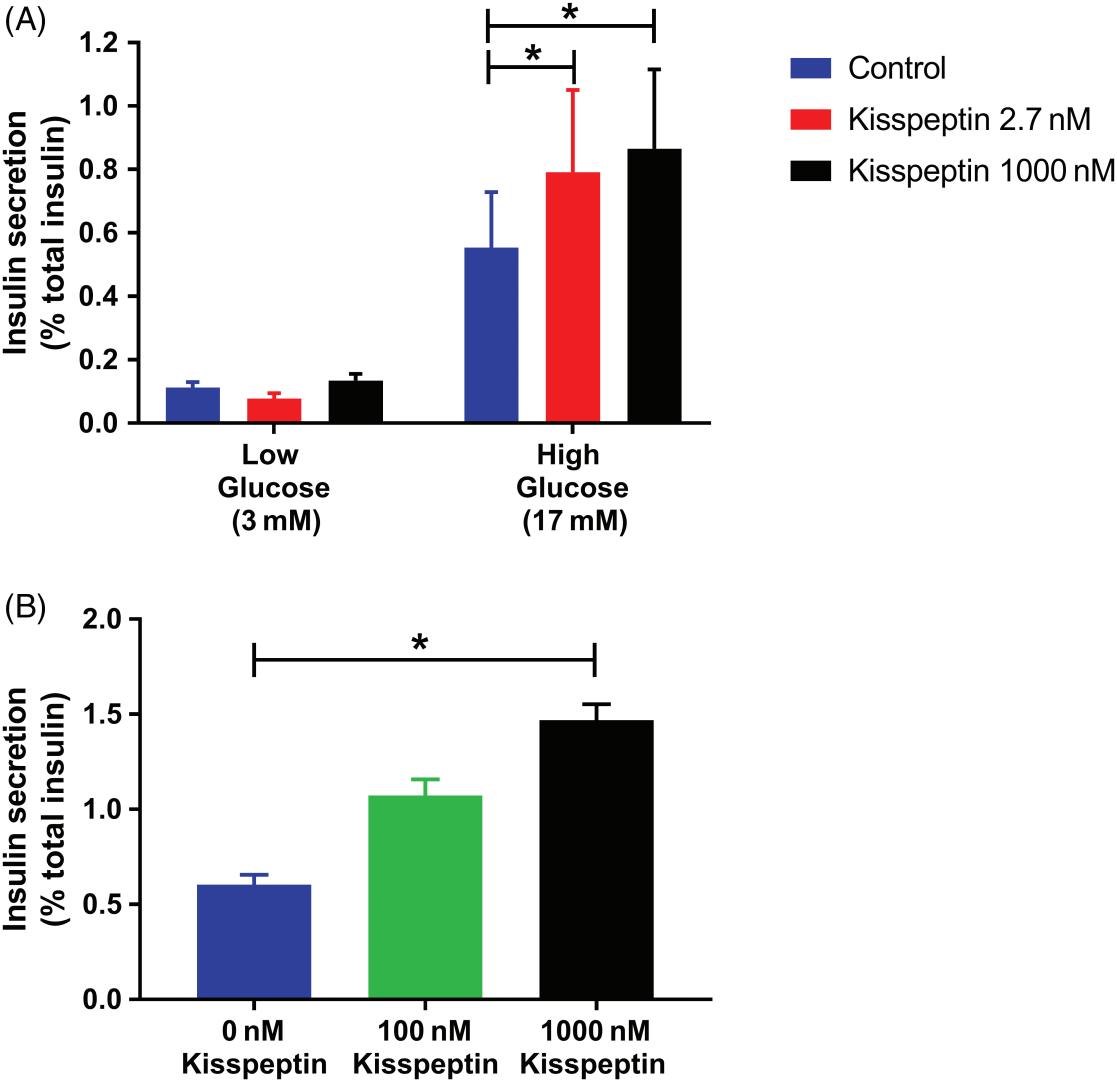
Kisspeptin enhances GSIS in cultured human donor islets and a human pancreatic β‐cell line. A, Insulin secretion was measured in human islet preparations (n = 6 donors) at low (3 mM) and high (17 mM) concentrations of glucose in the presence of increasing concentrations of kisspeptin (0 nM [blue bars], 2.7 nM [red bars] or 1000 nM [black bars]). Insulin secretion was normalized to percentage of total secretion; * p < 0.05 (Friedman test with Dunn's multiple comparison tests). B, Insulin secretion in cultured EndoC‐βH1 cells (n = 3 experiments) was measured at high glucose concentrations (15 mM) in the presence of increasing amounts of kisspeptin (0 nM [blue bars], 100 nM [green bars] or 1000 nM [black bars]). Insulin secretion was normalized to percentage of total secretion; * p < 0.05 (two‐way ANOVA with Dunnett's multiple comparison tests). Data presented as mean ± SEM

To assess the effect of kisspeptin on GSIS in vitro, human islets from six donors were incubated with 3 and 17 mM glucose with a range of concentrations of kisspeptin (0, 2.7 and 1000 nM kisspeptin). In donor human islets, kisspeptin enhanced insulin secretion (Figures 2A and S2), providing key mechanistic evidence that kisspeptin can stimulate GSIS by direct action on β‐cells. Crucially, kisspeptin stimulated human islet insulin secretion (in the presence of elevated glucose levels, Figure 2A) at similar kisspeptin concentrations to the circulating kisspeptin levels obtained in our human in vivo IVGTT studies above (i.e. 2‐3 nM, Figure 1B). Incubation with kisspeptin increased the insulin stimulation index (insulin stimulation index: 2.7 nM kisspeptin 11.08 ± 3.39 vs. 0 nM 5.09 ± 1.18, p < 0.01; 1000 nM 6.83 ± 1.84 vs. 0 nM 5.09 ± 1.18, p = 0.0866), but did not affect the insulin content of the donor islet preparations (Figure S3).

To further evaluate the effect of kisspeptin on GSIS in vitro, EndoC‐βH1 cells (a validated human β‐cell line, which retains many of the features of human pancreatic β‐cells)^34^, ^35^ were incubated with 15 mM glucose and kisspeptin concentrations, which have been used previously to investigate the effects of kisspeptin on GSIS in vitro (0, 100 and 1000 nM kisspeptin).^8^, ^10^, ^13^ Similar to the donor human islets, kisspeptin increased insulin secretion from EndoC‐βH1 cells in a dose‐dependent manner (Figure 2B).

### 3.3 Kisspeptin alters the pattern of specific serum metabolites in humans

**Figure 3:**
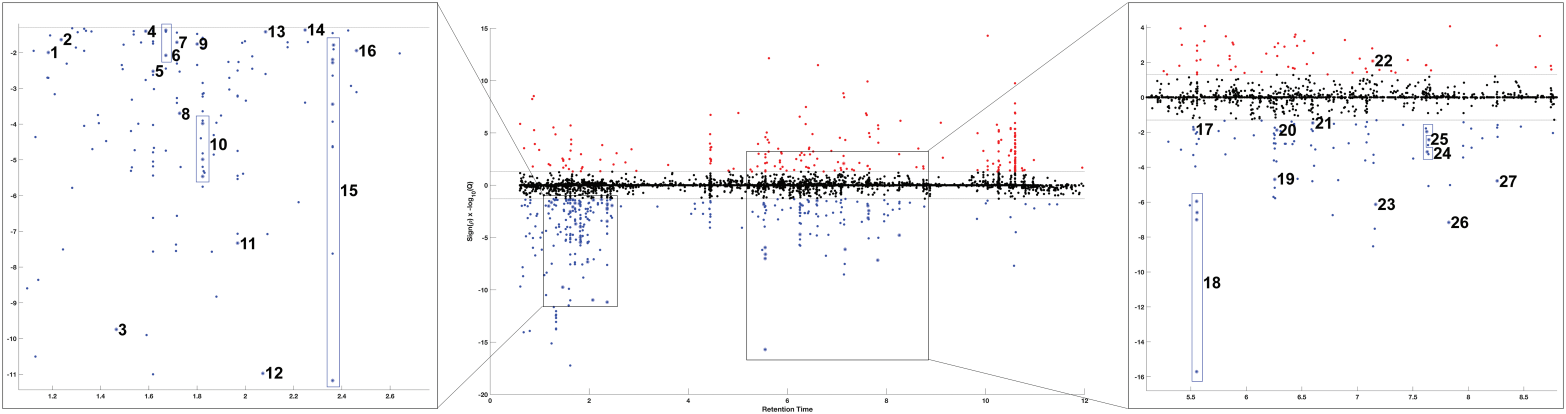
Kisspeptin modulates the metabolic profile in healthy men. Manhattan plot of the 5200 lipid species detected (in serum samples from 15 healthy male volunteers) by UPLC‐MS. 392 features showing a change over time significantly associated with kisspeptin administration are coloured red (increasing) or blue (decreasing). Statistical significance was determined based on a Q value threshold of 5%, where Q represents the local FDR‐corrected value of the appropriate linear mixed effect model estimates. Features successfully annotated are indicated on the plot as follows: 1, LPI(20:4); 2, LPC(0:0/14:0); 3, LPI(18:1); 4, LPE(0:0/18:2); 5, LPC(18:2/0:0); 6, LPE(18:2/0:0); 7, LPC(0:0/16:0); 8, CAR(20:3); 9, LPC(20:3/0:0); 10, LPC(16:0/0:0); 11, LPC(18:1/0:0); 12, Sphinganine(d18:0); 13, LPC(20:2/0:0); 14, LPC(0:0/18:0); 15, LPC(18:0/0:0) ; 16, LPC(20:1/0:0); 17, PC(18:2/18:2); 18, SM(d18:1/16:0); 19, PC(16:0/16:0); 20, SM(d18:1/18:0); 21, PC(18:0/18:2); 22, SM(d18:2/24:1); 23, SM(d18:2/22:0); 24, SM(d18:1/24:1); 25, SM(d18:1/22:0); 26, SM(d18:2/24:0); 27, SM(d18:1/24:0). Where LPI: lysophosphatidylinositol; LPC: lysophosphocholine; PC: phosphocholine; SM: sphingomyelin; LPE: lysophosphatidylethanolamine; CAR: Fatty acyl carnitine (see also Table S4)

To provide further insights into the metabolic effects of kisspeptin, blood samples from 15 volunteers during 30 1 nmol kg^−1^ h^−1^ kisspeptin infusions and 30 vehicle infusions were taken at T = −15 minutes (i.e. pre‐infusion) and at T = 45 minutes (i.e. when kisspeptin had reached steady state but prior to the glucose load) (Figure 1A), and serum was analysed using UPLC‐MS. Linear mixed effect modelling^27^ was used to identify patterns of serum metabolites, which changed significantly between baseline (T = −15 minutes) and steady state (T = 45 minutes) during kisspeptin but not vehicle administration. The profiles of numerous small molecules (Figure S4) and lipid species (Figure 3) were altered, including lysophosphatidylcholine (LPC 18:2), which was reduced by kisspeptin administration and has previously been shown to be negatively correlated with insulin secretion.^36^ All annotated metabolites that significantly changed during kisspeptin administration (but not vehicle administration) have been collated in Table S4.

### 3.4 Kisspeptin does not affect appetite or food intake in humans

**Figure 4:**
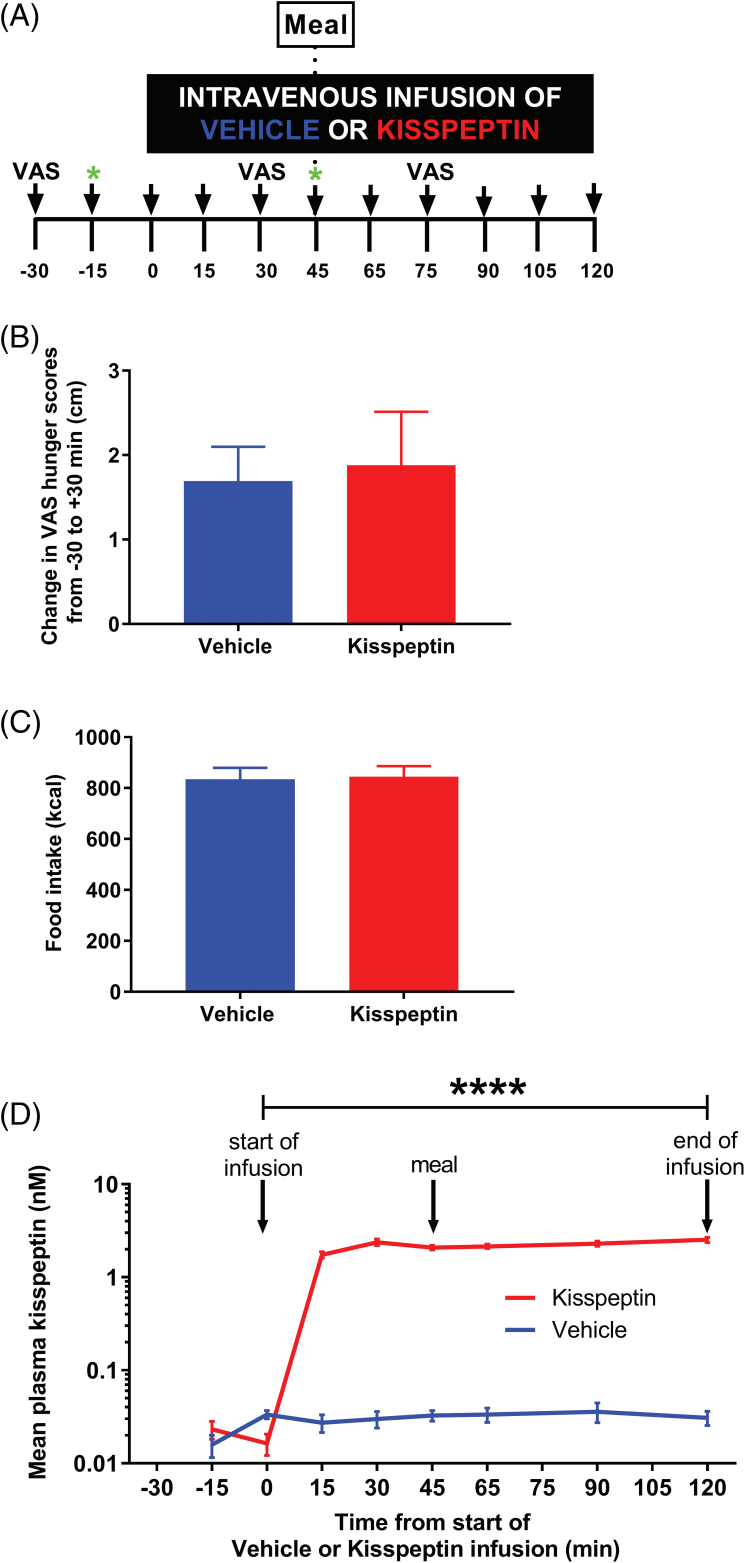
Kisspeptin administration does not affect appetite or food intake in healthy men. A, Following an overnight fast, 15 healthy men (age: 25.2 ± 1.1 years; BMI: 22.3 ± 0.5 kg m^−2^) were administered 1 nmol kg^−1^ h^−1^ kisspeptin or rate‐matched vehicle for 120 minutes, in random order. At T = 45 minutes, participants were given an ad libitum meal (which was eaten over a maximum of 20 minutes). Blood sampling was performed regularly (black arrows), with samples for metabolic profiling collected at T = −15 minutes and T = 45 minutes (green stars). Visual analogue scores (VAS) for volunteer‐reported hunger were recorded at T = −30 minutes, T = 30 minutes and T = 75 minutes. B, Mean change in pre‐meal volunteer‐reported hunger scores from T = −30 minutes to T = 30 minutes, as measured with VAS, were similar during kisspeptin (red bar) and vehicle (blue bar) administration; p = 0.8120 kisspeptin vs. vehicle (paired t‐test). C, Mean number of kcal ingested was similar during kisspeptin (red bar) and vehicle (blue bar) administration; p = 0.7178 kisspeptin vs. vehicle (paired t‐test). D, During MMTT, mean plasma kisspeptin levels were higher during kisspeptin compared with vehicle administration as expected; **** p < 0.0001 kisspeptin vs. vehicle (GEE). Data are presented as mean ± SEM; n = 15 per group

To assess the effect of kisspeptin on appetite and food intake in humans, 1 nmol kg^−1^ h^−1^ kisspeptin infusion, or rate‐matched vehicle infusion, was administered to 15 healthy young men. Participants were asked to record hunger and appetite on a visual analogue scale (VAS, range 0‐10 cm) at several timepoints during the infusion and were given an ad libitum meal at T = 45 minutes (i.e. when kisspeptin levels were at steady state) (Figure 4A). Self‐reported hunger was not significantly different during kisspeptin or vehicle administration (increase in hunger VAS scores from T = −30 minutes (baseline) to T = 30 minutes (i.e. 30 minutes after the start of the infusion): kisspeptin 1.88 ± 0.63 cm vs. vehicle 1.69 ± 0.40 cm, p = 0.8120) (Figure 4B). Hunger VAS scores from T = 30 minutes (pre‐meal) to T = 75 minutes (post‐meal) decreased similarly during kisspeptin and vehicle administration (pre‐meal to post‐meal change in VAS: kisspeptin −6.02 ± 0.56 cm vs. vehicle −5.6 ± 0.49 cm, p = 0.5183).

Furthermore, there was no difference in the mean kilocalories (kcal) consumed during the ad libitum meal during kisspeptin and vehicle administration (kisspeptin 844.7 ± 160.9 kcal vs. vehicle 834.4 ± 174.8 kcal, p = 0.7178) (Figure 4C).

As expected, following ad libitum meal ingestion, mean glucose levels achieved were lower than during IVGTT post‐glucose load (mean glucose level: IVGTT 9.72 ± 0.49 mM vs. MMTT 6.62 ± 0.20 mM, p < 0.05), with no associated effect of kisspeptin administration on insulin levels (Figure S5A), glucose levels (Figure S5B) or MMTT‐DI (kisspeptin 696.3 ± 159.1 units vs. vehicle 671.9 ± 145.6 units, p = 0.6286).

During kisspeptin administration, plasma kisspeptin levels were elevated (Figure 4D), which resulted in elevated LH levels (Figure S5C) and no significant change in testosterone (Figure S5D), similar to those from IVGTTs.

## 4 DISCUSSION

This is the first study investigating the effect of kisspeptin on β‐cell function and appetite in humans in vivo. We demonstrate that the reproductive hormone kisspeptin enhances GSIS in humans in vivo without affecting insulin sensitivity. Our in vitro data show a direct effect of kisspeptin on β‐cells, which are known to possess abundant kisspeptin receptors.^8^ Furthermore, kisspeptin modulates serum metabolites in humans, but does not influence appetite or food intake. These data indicate that kisspeptin plays a role in glucose homeostasis in humans, and therefore is a hormonal mediator linking reproductive and metabolic systems.

Kisspeptin administration increased insulin secretion and disposition index during IVGTT but not during MMTT. The glucose concentrations were more markedly elevated (as expected) during the IVGTT (mean peak glucose 14.01 mM, Figure 1E) compared to the MMTT (mean peak glucose 7.48 mM, Figure S5B). Therefore, this suggests that kisspeptin increases insulin release only when ambient glucose concentrations are high in humans in vivo. In keeping with this, our data show that in human islets in vitro, kisspeptin increases insulin secretion at 17 mM but not at 3 mM glucose (Figure 2A). This is also consistent with previous data in human islets in vitro, which show that kisspeptin stimulates insulin release at higher ambient glucose concentrations (20 vs. 3 mM).^8^, ^12^

However, there are differing reports of the effects of kisspeptin on insulin secretion from animal islets. Kisspeptin, at doses ranging from 0.1‐1000 nM, has been reported to increase GSIS in mouse, rat and pig islets, when the prevailing glucose concentrations were ≥ 10 mM.^8^, ^10^, ^12^, ^37^ In contrast, other investigators have reported that kisspeptin, at doses ranging from 0.1‐1000 nM, inhibits GSIS in a dose‐dependent manner in mouse and rat islets/perfused pancreata.^13^, ^14^, ^38^ The lack of consensus in the literature may be due to methodological differences in the experiments and/or species differences, which are often observed between animal and human islet preparations.^39^ Our human data in a range of kisspeptin doses in vitro and at 1 nmolkg^−1^ h^−1^ in vivo in the current study do, however, demonstrate positive effects of kisspeptin on GSIS. Nevertheless, given that only one concentration of kisspeptin was used in our in vivo study, further studies are required to determine the effects of kisspeptin on GSIS *in vivo* across a dose spectrum.

It is interesting to note that the increase in post‐load IVGTT insulin concentrations produced by kisspeptin administration was not accompanied by a change in glucose levels. This could suggest some loss of sensitivity to insulin. However, S_i_, as measured with the minimal model, was not reduced. Additionally, fasting insulin concentrations, which rise with reductions in S_i_,^40^ were unaffected by kisspeptin. Another explanation for the maintenance of glucose levels could be counter‐regulatory cortisol secretion. However, we measured cortisol levels in the IVGTT samples and there was no significant difference in cortisol during kisspeptin administration compared to vehicle (Figure S1F). Overall, it appears that although kisspeptin enhanced insulin secretion, in healthy individuals this did not result in altered glucose levels, but this would be interesting to investigate in patients with abnormal glucose homeostasis such as in diabetes.

Testosterone has also been shown to increase insulin secretion from isolated islets.^41^ However, the effects of kisspeptin administration on metabolism during our study were not confounded by altered circulating testosterone levels as serum testosterone did not rise during the time‐period of this study, because more prolonged kisspeptin administration is required to produce elevations in testosterone levels.^33^

The gut hormones, GLP‐1, PYY and glucagon have key roles in glucose homeostasis. Additionally, in rodent studies, GLP‐1 has been shown to alter hypothalamic kisspeptin expression and neuronal activity,^42^, ^43^ and in mice glucagon stimulates hepatic kisspeptin production to alter GSIS.^13^ In our study, there was no difference in circulating GLP‐1, PYY or glucagon levels following intravenous glucose during kisspeptin administration compared to vehicle (Figure S1C‐E). This is consistent with previous data which show that intravenous glucose administration alone does not activate the incretin response mediated by gut hormones.^44^ Furthermore, the above studies suggest that GLP‐1 and glucagon may act upstream of kisspeptin, whereas in our study we directly administered kisspeptin, which may act downstream of GLP‐1 and glucagon.

The participants were fasted during the IVGTTs, and fasting has been shown to reduce hypothalamic kisspeptin expression.^45^ However, as shown in Figure 1B, kisspeptin administration significantly raised plasma kisspeptin levels, and therefore would be sufficient to overcome the effect of suppression of endogenous kisspeptin by fasting. Furthermore, kisspeptin administration has been shown to increase GSIS in both fasted and fed monkeys.^11^

Metabonomics, the identification and analysis of metabolites in biological fluids, is an emerging field of study which provides a non‐biased methodology to identify novel pathways to guide further research. To provide further insights into the metabolic effects of kisspeptin, we compared the distribution of metabolites in serum samples taken pre‐kisspeptin administration (T = −15 minutes) to those taken when plasma kisspeptin levels had reached steady state (T = 45 minutes) and equivalent vehicle timepoints. Importantly, samples were collected before the glucose loads to prevent this from confounding the results. We demonstrate for the first time that kisspeptin modulates serum lipids and small molecules in humans. Several of these metabolites include classes of lipids (i.e. lysophosphatidylcholines, phosphocholines and sphingomyelins), which have been shown to be associated with insulin secretion.^36^ This provides further evidence of kisspeptin's modulation of human metabolism, which (with further study) may provide important mechanistic data.

The principal limitation to the metabolite analysis approach used in the present study is that the measurements taken for each chemical species do not yield absolute quantities. Rather, they provide values that are proportional to the concentration of chemicals within the biological fluid, which are only useful in relative comparison within the experiment rather than in comparison to established concentration ranges. We have accounted for this limitation through the use of linear mixed effect models which rely solely on the differences observed in the relative abundance of individual metabolites to generate patterns of statistical significance. This allows the determination of significance and magnitude of metabolic changes.

In light of emerging evidence for neuroanatomical and functional connections between kisspeptin and key appetite‐regulating neurones in the hypothalamus,^15^, ^16^ and animal data which suggest a role for kisspeptin in energy homeostasis,^21^ we investigated the effect of kisspeptin on appetite in healthy men. Our data demonstrate kisspeptin had no effect on appetite and food intake in men. This is in keeping with rodent data showing that male *kiss1r* knockout mice have unaltered food intake.^21^

Our findings have pharmacological and potential therapeutic relevance as the plasma kisspeptin levels achieved in this study, which enhanced GSIS, are similar to those required to restore LH pulsatility in women with hypothalamic amenorrhea^3^ and trigger oocyte maturation in in vitro fertilisation protocols.^2^ During IVGTTs, pharmacological kisspeptin administration increased insulin secretion and increased disposition index (IVGTT‐DI). IVGTT‐DI quantifies the ability of the β‐cell to counter insulin resistance,^32^ with lower baseline IVGTT‐DI values independently predicting conversion from normal glucose tolerance or impaired glucose tolerance to type 2 diabetes within 5 years.^32^ Therefore, our finding that kisspeptin increases GSIS and IVGTT‐DI shows metabolically the beneficial effects of kisspeptin.

This is especially important as kisspeptin‐based treatments are currently being developed to treat reproductive disorders^2^, ^3^, ^4^, ^5^ and such treatments may therefore have additional potentially beneficial metabolic therapeutic applications. Our finding that kisspeptin administration also improves GSIS suggests that kisspeptin could have a dual therapeutic role in patients with diabetes to improve hypogonadism^33^ as well as enhance insulin release, specifically only when hyperglycaemia occurs (thereby potentially avoiding the risks of hypoglycaemia associated with other diabetes treatments). Thus, our study lays the foundation for future studies exploring the effect of kisspeptin in the treatment of diabetes. Furthermore, both kisspeptin‐54 and the smaller fragment, kisspeptin‐10 (which may be produced by enzymatic breakdown) have been shown to enhance GSIS in vivo in humans in this study and in vivo in monkeys,^11^ respectively. Therefore, metabolically active breakdown products of kisspeptin may serve to potentiate the metabolically beneficial effects of administered kisspeptin.

Our data demonstrating that pharmacological elevation of circulating kisspeptin levels results in a significant increase in GSIS in humans in vivo may also have potential physiological relevance. The circulating kisspeptin levels achieved by pharmacological administration of kisspeptin during this study are also observed in humans physiologically during pregnancy due to placental kisspeptin production (1‐10 nM).^46^ Therefore, the higher circulating kisspeptin levels during normal pregnancy may play a physiological role to enhance insulin secretion to protect the mother and foetus from increasing glucose levels and the development of gestational diabetes mellitus. In keeping with this hypothesis, pregnant women with diabetes have lower kisspeptin levels than pregnant women without diabetes.^47^ In addition, previous rodent work has shown that selective ablation of the kisspeptin receptor, *kiss1r*, from pancreatic islet β‐cells in pregnant mice results in impaired glucose tolerance and reduced insulin secretion.^48^ Together these data suggest that elevated kisspeptin during pregnancy may play a positive physiological role in glucose homeostasis to protect against the development of gestational diabetes mellitus.

In summary, reproduction and metabolism are fundamental and interdependent aspects of mammalian physiology. This comprehensive study demonstrates that administration of the reproductive hormone kisspeptin to humans significantly increases GSIS in vivo and in vitro via actions on pancreatic islet cells, with associated alterations in serum metabolites associated with insulin secretion. We also show that kisspeptin does not affect appetite or food intake in healthy men.

This is the first human in vivo report of the effect of kisspeptin on β‐cell function, metabolites and appetite, which is important for our understanding of the links between reproduction and metabolism in humans, as well as the ongoing development of kisspeptin‐based treatments for common reproductive and potential metabolic disorders.

## Supporting information


**File S1.** Supplementary Methods, Tables and Figures.
**Table S1.** Details of donor human islets.
**Table S2.** Standard mixtures.
**Table S3.** Gradient conditions for the reverse phase lipid separation.
**Table S4.** Metabolites modulated by kisspeptin in healthy young men.
**Figure S1.** Reproductive and Gut hormone levels during IVGTT.
**Figure S2.** Insulin secretion from six individual human donor islets incubated with different kisspeptin concentrations.
**Figure S3.** Insulin content from six individual human donor islets incubated with different kisspeptin concentrations.
**Figure S4.** Kisspeptin modulates small molecules in humans.
**Figure S5.** Reproductive hormone, insulin and glucose levels during MMMT.
