# Supplementary material for: The effects of kisspeptin on β‐cell function, serum metabolites and appetite in humans
Source: Diabetes Obes Metab. 2018 Aug 16;20(12):2800–10. doi: 10.1111/dom.13460 (PMC6282711; doi:10.1111/dom.13460)
Supplement: Supplementary file 1 — File S1. Supplementary Methods, Tables and Figures. Table S1. Details of donor human islets. Table S2. Standard mixtures. Table S3. Gradient conditions for the reverse phase lipid separation. Table S4. Metabolites modulated by kisspeptin in healthy young men. Figure S1. Reproductive and Gut hormone levels during IVGTT. Figure S2. Insulin secretion from six individual human donor islets incubated with different kisspeptin concentrations. Figure S3. Insulin content from six individual human donor islets incubated with different kisspeptin concentrations. Figure S4. Kisspeptin modulates small molecules in humans. Figure S5. Reproductive hormone, insulin and glucose levels during MMMT. [file DOM-20-2800-s001.docx]

**Supplementary Methods**

*Metabolite profiling*

**QUALITY CONTROL (QC) SAMPLES**

QC samples were injected at regular intervals throughout the run and used to support the analytical quality assessment of the data. Details of the two types of QC samples employed (long term reference, LTR, and study reference, SR) and the use of SR dilution series have been reported in Lewis et al. 2016^26^. To create the serum LTR, 10L of bulk serum were purchased from Seralab, homogenised, and aliquoted for long term storage.

Mixtures of authentic reference materials (Table S2) were added to SR and LTR samples to monitor data quality during acquisition. LTR and SR samples were mixed with assay-specific method reference (MR) mixtures in a 5:1 ratio (QC/MR) for hydrophilic interaction liquid chromatography (HILIC), and in a 1:4 ratio (QC/MR) for the reversed-phase chromatography (RPC).

**SAMPLE SORTING, FORMATTING, AND ALIQUOTTING**

Serum samples were stored at -80°C and remained frozen while being sorted according to the analysis order established, avoiding confounding critical clinical variables with analytical run order effects. After sorting and overnight thawing at 4°C, sets of 80 samples were formatted in 96-deep-well polypropylene plates (2mL, Eppendorf). Columns 11 and 12 of the plates were left empty for addition of pooled QC samples. The plates were sealed with silicone cap mats and centrifuged at 3486×g for 10mins at 4°C (Eppendorf 5810R centrifuge; rotor A-2-DWP-AT). Following centrifugation, serum samples were aliquoted and solid fat particulates, when observed, were removed by adhesion to a clean pipette tip. Supernatant was then aspirated and dispensed using an eight-channel 15-1200µL pipette (Eppendorf Xplorer Plus) to individual 96-well polypropylene plates (Eppendorf) for HILICRPC assay-specific sample preparation, as well as for pooling to create the SR sample. All plates were sealed with silicone cap mats and returned to -80°C storage.

**SERUM UPLC-MS ANALYSES**

HILIC profiling

Serum samples were thawed at 4°C for 2h. Subsequently, samples were diluted 1:1 with H_2_O and HILIC internal standards (IS) (Table S2). Three parts of acetonitrile were then added to one part of diluted sample for protein precipitation. Each plate was heat sealed (Thermo-Seal heat sealing foil sheets) using a ALPS 50 V-Manual Heat Sealer (Thermo Scientific) prior to mixing at 1400rpm for 2h at 4°C (MixMate, Eppendorf). The plates were then centrifuged for 10mins at 3486×g and 4°C to separate the homogenous supernatant from the precipitated protein. The supernatant (125µL) was aspirated and dispensed into 96-well polypropylene plates (Eppendorf), heat sealed, and centrifuged for 5mins at 3486×g and 4°C prior analysis. Details of the HILIC UPLC method and MS parameters have been reported in Lewis et al. 2016^26^.

RPC profiling

Serum samples were thawed at 4°C for 2h. Subsequently, samples were prepared for lipid analysis by dilution with H_2_O (1:1 v/v) and further addition of four parts of isopropanol containing a mixture of reference standards (Table S3) to one part of diluted sample for protein precipitation. Plates were heat sealed prior to mixing at 1400rpm for 2h at 4°C and subsequently centrifuged for 10mins at 3486×g and 4°C to separate the homogenous supernatant from the precipitated protein. The supernatant (125µL) was aspirated and dispensed into 96-well polypropylene plates (Eppendorf), heat sealed, and centrifuged for 5mins at 3486×g and 4°C prior analysis. Prepared samples (2µL) were injected in the chromatographic system using full loop mode (5× overfill). Lipidomic profiling was conducted using a 2.1×100mm BEH C8 column, thermostatted at 55°C. Solvent A consisted of a 50:25:25 mixture of H_2_O:ACN:IPA with 5mm ammonium acetate, 0.05% acetic acid, and 20µM phosphoric acid (to improve the peak shape of some phospholipid species). Solvent B consisted of 50:50 ACN:IPA with 5mm ammonium acetate, 0.05% acetic acid. The initial conditions were 99:1 A:B at a flow rate of 0.6mL.min^-1^. The gradient elution program is shown in Table S3.

The mass spectrometry parameters for lipid analysis were set as follows: capillary voltage 1.5/2kV, sample cone voltage 20/25V, source temperature 120°C, desolvation temperature 600°C, desolvation gas flow 1000L.h^-1^, and cone gas flow 150L.h^-1^. Data were collected in centroid mode with a scan range of 50-2000m.z^-1^ and a scan time of 0.07-0.15s. For mass accuracy, LockSpray mass correction was performed using a 200pg.μL^-1^ leucine enkephalin solution (m.z^-1^ 556.2771 in ESI+) in 50:50 H_2_O:ACN solution at a flow rate of 10μL.min^-1^. Lockmass scans were collected every 60s and averaged over 3 scans.

**Supplementary Tables**

**Table S1: Details of donor human islets**

| DONOR | SOURCE |
| --- | --- |
| Healthy human pancreatic islets  (F, 46y, BMI 35, no cause of death provided) | Oxford Consortium for Islet Transplantation, University of Oxford  https://www.nds.ox.ac.uk/research/oxcit |
| Healthy human pancreatic islets  (F, 66y, BMI 18.5, Hba1c 6.1% (43.2mmol/mol), no cause of death provided) | The MacDonald Islet Biology Laboratory, University of Alberta  http://www.bcell.org |
| Healthy human pancreatic islets  (M, 84y, BMI 23.2, death due to cerebral haemorrhage) | Department of Endocrinology and Metabolism, University of Pisa  https://www.unipi.it/index.php/english |
| Healthy human pancreatic islets  (M, 56y, BMI 32, death due to anoxia) | IRCCS San Rafaele Scientific Institute, Milan  http://www.hsr.it/research |
| Healthy human pancreatic islets  (F, 75y, BMI 28.8, no cause of death provided) | Clinical Islet Laboratory and Clinical Islet Transplant Program, University of Alberta, Edmonton, Alberta, Canada  http://www.islet.ca |
| Healthy human pancreatic islets  (M, 44y, BMI 36, no cause of death provided) | Oxford Consortium for Islet Transplantation, University of Oxford  https://www.nds.ox.ac.uk/research/oxcit |

**Table S2: Standard mixtures**

| *No.* | *Standard* | *Supplier & Part number* |
| --- | --- | --- |
| HILIC method reference mixture | | |
| 1 | Phenylalanine-^13^C_9_,^15^N | Sigma, 608017 |
| 2 | Adenine-2d_1_ | CDN isotopes, D-6291 |
| 3 | Taurine-^15^N | Sigma, 605956 |
| 4 | Creatine-d_3_.H_2_O | Sigma, 616249 |
| 5 | Arginine-^13^C_6_ | Sigma, 643440 |
| 6 | Tryptophan-d_5_ | Sigma, 615862 |
| 7 | Uracil-2-^13^C,^15^N_2_ | Sigma, 608459 |
| HILIC internal standards mixture | | |
| 1 | N-Benzoyl-d5-glycine | CDN isotopes, D-5588 |
| 2 | Adenosine-2-d-1 | CDN isotopes, D-1827 |
| Lipid method reference/internal standards mixture | | |
| 1 | LPC9 | Avanti, 855276P |
| 2 | PC11 | Avanti, 850330P |
| 3 | C17 | Sigma, H3500 |
| 4 | PG15 | Avanti, 840446P |
| 5 | PE15 | Avanti, 850704P |
| 6 | PS17 | Avanti, 840028P |
| 7 | PA17 | Avanti, 830856P |
| 8 | Cer17 | Avanti, 860517P |
| 9 | DG19 | Sigma, 68633 |
| 10 | PC23 | Avanti, 850372P |
| 11 | TG15 | Sigma, T4257 |
| 12 | TG17 | Sigma, T2151 |

**Table S3: Gradient conditions for the reverse phase lipid separation**

| *Step* | *Time (mins)* | *Flow (mL.min^-1^)* | *% A* |
| --- | --- | --- | --- |
| 1 | Initial | 0.6 | 99.0 |
| 2 | 0.10 | 0.6 | 99.0 |
| 3 | 2.00 | 0.6 | 70.0 |
| 4 | 11.50 | 0.6 | 10.0 |
| 5 | 12.00 | 1.0 | 0.1 |
| 6 | 12.5 | 1.0 | 0.1 |
| 7 | 12.55 | 0.9 | 35.0 |
| 8 | 12.65 | 0.8 | 70.0 |
| 9 | 12.75 | 0.7 | 99.0 |
| 10 | 12.95 | 0.6 | 99.0 |
| 11 (sample load) | 15.00 | 0.6 | 99.0 |

**Table S4: Metabolites modulated by kisspeptin in healthy young men**

| **ASSAY** | **METABOLITE ANNOTATION** | **RETENTION TIME** | **MASS** | **ION** | **FOLD-CHANGE** | **FDR-CORRECTED P-VALUE** |
| --- | --- | --- | --- | --- | --- | --- |
| **HILIC (+)** | 5'-Methylthioadenosine* | 1.34 | 298.097 | [M+H]+ | -11.687 | 6.12E-03 |
| **RPC (+)** | CAR(20:3) | 1.73 | 450.357 | [M+H]+ | -10.317 | 2.01E-04 |
|  | LPC(0:0/14:0) | 1.24 | 450.297 | [M+H-H2O]+ | -5.142 | 2.31E-02 |
|  | LPC(0:0/16:0) | 1.72 | 991.672 | [2M+H]+ | -29.666 | 1.95E-02 |
|  | LPC(0:0/18:0) | 2.25 | 506.360 | [M+H-H2O]+ | -4.035 | 4.30E-02 |
|  | LPC(16:0/0:0) | 1.82 | 1013.654 | [2M+Na]+ | -20.091 | 1.03E-05 |
|  |  | 1.82 | 1029.628 | [2M+K]+ | -18.204 | 1.04E-04 |
|  |  | 1.82 | 991.673 | [2M+H]+ | -15.221 | 3.44E-06 |
|  | LPC(18:0/0:0) | 2.36 | 1047.735 | [2M+H]+ | -22.308 | 3.61E-04 |
|  |  | 2.36 | 281.662 | [M+H+K]2+ | -14.191 | 6.68E-12 |
|  |  | 2.36 | 546.353 | [M+Na]+ | -4.504 | 6.42E-03 |
|  |  | 2.36 | 562.327 | [M+K]+ | -4.457 | 5.24E-03 |
|  |  | 2.37 | 524.372 | [M+H]+ | -4.310 | 1.63E-02 |
|  | LPC(18:1/0:0) | 1.97 | 280.654 | [M+H+K]2+ | -12.906 | 4.68E-08 |
|  | LPC(18:2/0:0) | 1.62 | 558.296 | [M+K]+ | -7.288 | 3.01E-03 |
|  | LPC(20:1/0:0) | 2.46 | 588.350 | [M+K]+ | -4.996 | 1.14E-02 |
|  | LPC(20:2/0:0) | 2.08 | 570.354 | [M+Na]+ | -4.867 | 3.83E-02 |
|  | LPC(20:3/0:0) | 1.80 | 528.343 | [M+H-H2O]+ | -5.942 | 1.74E-02 |
|  | LPE(0:0/18:2) | 1.59 | 516.252 | [M+K]+ | -5.573 | 4.01E-02 |
|  | LPE(18:2/0:0) | 1.67 | 500.275 | [M+Na]+ | -6.601 | 8.39E-03 |
|  |  | 1.67 | 516.249 | [M+K]+ | -7.213 | 4.24E-02 |
|  | LPI(18:1) | 1.46 | 637.275 | [M+K]+ | -21.693 | 1.80E-10 |
|  | LPI(20:4) | 1.18 | 659.259 | [M+K]+ | -10.229 | 1.01E-02 |
|  | PC(16:0/16:0) | 6.26 | 1468.141 | [2M+H]+ | -12.754 | 1.99E-05 |
|  | PC(18:0/18:2) | 6.60 | 1572.201 | [2M+H]+ | -5.747 | 3.41E-02 |
|  | PC(18:2/18:2) | 5.53 | 1564.131 | [2M+H]+ | -16.965 | 1.45E-02 |
|  | SM(d18:1/16:0) | 5.55 | 1406.147 | [2M+H]+ | -9.159 | 9.84E-08 |
|  |  | 5.55 | 1428.128 | [2M+Na]+ | -12.577 | 1.11E-06 |
|  |  | 5.55 | 1444.102 | [2M+K]+ | -17.946 | 1.97E-16 |
|  |  | 5.56 | 886.642 | [M+GrHd-H]+ | -6.413 | 2.51E-07 |
|  | SM(d18:1/18:0) | 6.27 | 1462.210 | [2M+H]+ | -10.474 | 1.33E-02 |
|  | SM(d18:1/22:0) | 7.64 | 1574.333 | [2M+H]+ | -10.965 | 3.71E-03 |
|  | SM(d18:1/24:0) | 8.26 | 1630.395 | [2M+H]+ | -17.461 | 1.65E-05 |
|  | SM(d18:1/24:1) | 7.63 | 1626.366 | [2M+H]+ | -8.409 | 1.09E-02 |
|  |  | 7.63 | 1648.343 | [2M+Na]+ | -13.131 | 7.57E-04 |
|  | SM(d18:2/22:0) | 7.16 | 1570.302 | [2M+H]+ | -16.009 | 7.43E-07 |
|  | SM(d18:2/24:0) | 7.82 | 1626.362 | [2M+H]+ | -19.305 | 6.89E-08 |
|  | SM(d18:2/24:1) | 7.14 | 610.590 | [M-GrHd-H2O+H]+ | 3.246 | 8.37E-03 |
|  | Sphinganine (d18:0) | 2.07 | 302.304 | [M+H]+ | -16.902 | 1.06E-11 |

Serum samples taken at T=-15mins and T=45mins (i.e. prior to IV glucose load/meal) underwent metabolite analysis by ultra-performance liquid chromatography-mass spectrometry, followed by linear mixed effect modelling to identify those metabolites which were significantly modulated by kisspeptin administration (but not vehicle). These metabolites were annotated based on accurate mass and tandem mass spectrometry (MS/MS) fragmentation match using LIPID MAPS online tools (for lipid species)^29^ or an in-house database constructed from analysis of authentic reference materials (for 5'-Methylthioadenosine). Where possible, the identity of assigned metabolites was confirmed by direct comparison of chromatographic and spectral qualities to authentic reference material including chromatographic spike-in (denoted by *). The HILIC (+) assay utilised hydrophilic interaction liquid chromatography and positive mode ion detection, while the RPC (+) assay utilised a reversed-phase chromatographic separation optimised for the separation of complex lipids and positive ion mode detection. GrHd: grouphead.

**Figure S1**

**Figure S2**

**Figure S3**

**Figure S4**


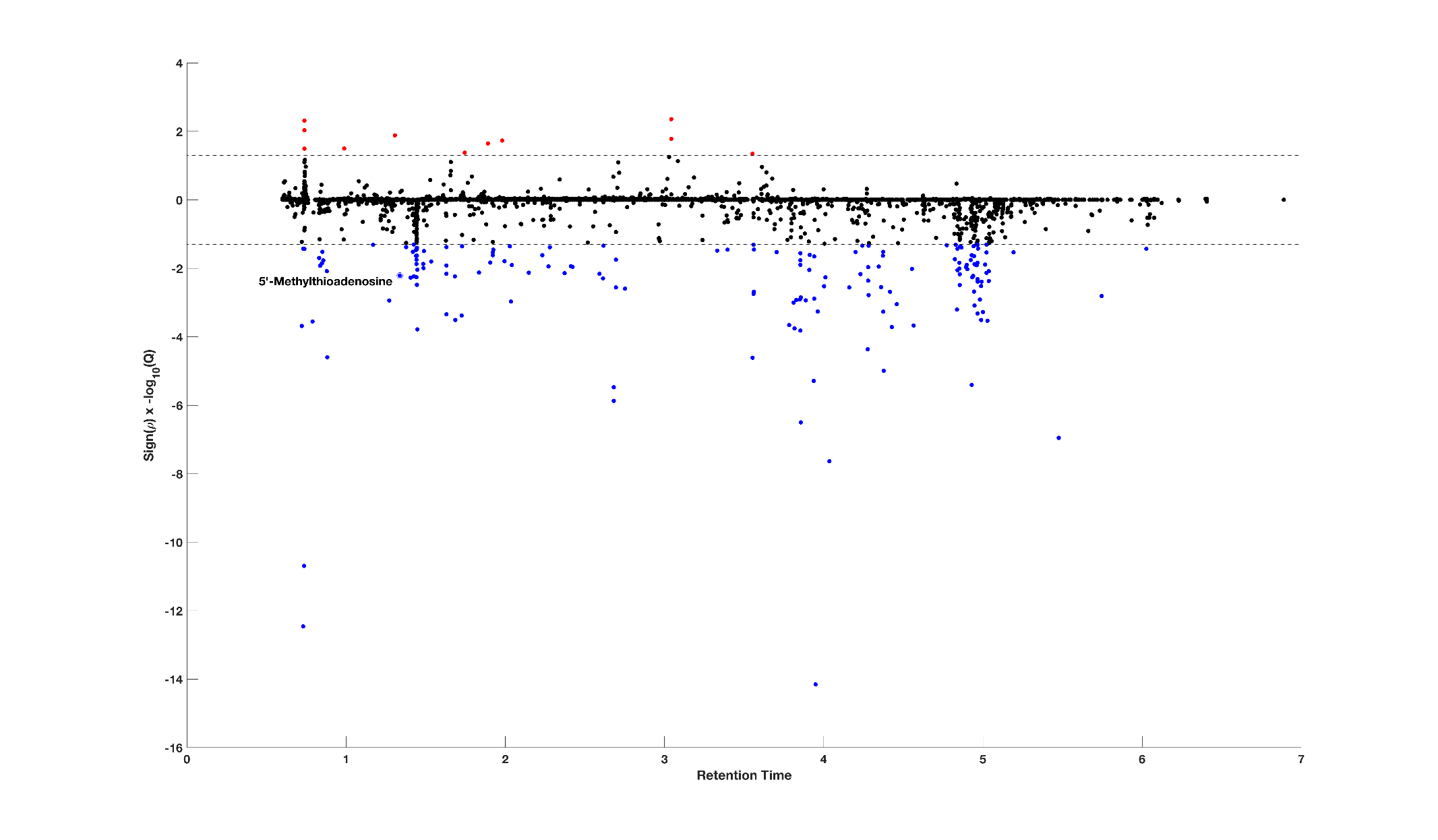


**Figure S5**

**Supplementary Figure Legends**

**Figure S1: Reproductive and Gut hormone levels during IVGTT**

**A**: Mean serum luteinising hormone (LH) levels during intravenous glucose tolerance test (IVGTT) were elevated during kisspeptin administration confirming peptide bioactivity. Data presented as mean±SEM. n=15 per group, ****p<0.0001 (GEE).

**B**: Mean serum testosterone levels during IVGTT were similar with kisspeptin and vehicle administration. Data presented as mean±SEM. n=15 per group. From start of kisspeptin infusion (T=0mins) to T=225mins p=0.3833 kisspeptin vs vehicle (GEE); from start of IVGTT (i.e. T=45mins) to T=225mins p=0.2386 kisspeptin vs vehicle (GEE).

**C**: Plasma glucagon-like peptide_7-36_ (GLP-1_7-36_) levels were similar during IVGTTs with kisspeptin and vehicle administration. Data presented as mean±SEM. n=15 per group. From T=0mins to T=225 mins p=0.5912 kisspeptin vs vehicle (GEE).

**D**: Plasma peptide-YY (PYY) levels were similar during IVGTTs with kisspeptin and vehicle administration. Data presented as mean±SEM. n=15 per group. From T=0mins to T=225mins p=0.5103 kisspeptin vs vehicle (GEE).

**E**: Plasma glucagon levels were similar during IVGTTs with kisspeptin and vehicle administration. Data presented as mean±SEM. n=15 per group. From T=0mins to T=225mins p=0.9459 kisspeptin vs vehicle (GEE).

**F**: Serum cortisol levels were similar during IVGTTs with kisspeptin and vehicle administration. Data presented as mean±SEM. n=15 per group. From T=0mins to T=225mins p=0.6650 kisspeptin vs vehicle (GEE).

**Figure S2 Insulin secretion from six individual human donor islets incubated with different kisspeptin concentrations**

**A-F**: Insulin secretion was measured in human islet preparations at low (3mM glucose) and high (17mM glucose) concentrations of glucose in the presence of different concentrations of kisspeptin (0nM (blue lines), 2.7nM (red lines) or 1000nM (black lines)). Insulin secretion was normalised to percentage of total secretion.

**Figure S3 Insulin content from six individual human donor islets incubated with different kisspeptin concentrations**

**A-F**: Insulin content was measured in human islet preparations in the presence of different concentrations of kisspeptin (0nM (blue bars), 2.7nM (red bars) or 1000nM (black bars)).

**Figure S4 Kisspeptin modulates small molecules in humans**

Manhattan plot of the small molecules detected (in serum samples from 15 healthy male volunteers) by ultra-performance liquid chromatography mass spectrometry (UPLC-MS). Features showing a change over time significantly associated with kisspeptin administration are coloured red (increasing) or blue (decreasing). Statistical significance was determined based on a Q value threshold of 5%, where Q represents the local FDR corrected value of the appropriate linear mixed effect model estimates. Features successfully annotated are indicated on the plot (see also Table S4).

**Figure S5 Reproductive hormone, insulin and glucose levels during MMMT**

**A**: Mean serum insulin levels during MMTT were similar during kisseptin compared to vehicle administration. Data presented as mean±SEM. n=15 per group. From T=45mins to T=120mins p=0.10 kisspeptin vs vehicle (multi-level linear regression).

**B**: Mean plasma glucose levels during MMTT were similar during kisspeptin and vehicle administration. Data presented as mean±SEM. n=15 per group. From T=45mins to T=120mins p=0.25 kisspeptin vs vehicle (multi-level linear regression).

**C**: Mean serum luteinising hormone (LH) levels during mixed meal tolerance test (MMTT) were elevated during kisspeptin administration confirming peptide bioactivity. Data presented as mean±SEM. n=15 per group, ****p<0.0001 (GEE).

**D**: Mean serum testosterone levels during MMTT were similar during kisspeptin and vehicle administration. Data presented as mean±SEM. n=15 per group. From start of kisspeptin infusion (T=0mins) to T=120mins p=0.4072 kisspeptin vs vehicle (GEE); from start of MMTT (T=45mins) to T=120mins p=0.2303 kisspeptin vs vehicle (GEE).
